# Novel Cyclovirus Identified in Broiler Chickens With Transmissible Viral Proventriculitis in China

**DOI:** 10.3389/fvets.2020.569098

**Published:** 2020-09-29

**Authors:** Tianxing Yan, Gen Li, Defang Zhou, Xiaoxia Yang, Liping Hu, Ziqiang Cheng

**Affiliations:** ^1^College of Veterinary Medicine, Shandong Agricultural University, Tai'an, China; ^2^College of Veterinary Medicine, Qingdao Agricultural University, Qingdao, China; ^3^Hospital of Shandong Agricultural University, Tai'an, China; ^4^Animal Epidemic Prevention and Control Center of Shandong Province, Jinan, China

**Keywords:** Cyclovirus, Circoviridae, transmissible viral proventriculitis, broiler chicken, diarrhea

## Abstract

In October 2018, an outbreak of transmissible viral proventriculitis (TVP) occurred in 30-day-old commercial broiler chickens on a farm in Weifang, China. TVP, an infectious viral disease characterized by runting and stunting, is associated with many viruses, and has a significant economic impact on the global poultry industry. TVP is diagnosed according to clinical symptoms, gross and histological lesions, and negative PCR results for pathogenic bacteria, avian leukosis virus subgroup J, Marek's disease virus, reticuloendotheliosis virus, infectious bursa disease virus, avian reovirus, chicken anemia virus, infectious bronchitis virus, chicken proventricular necrosis virus, gyrovirus 3 and chicken circovirus. To further detect the possible causative pathogens of TVP, we used PacBio third-generation sequencing to examine proventricular samples. A dominant abundance of the novel cyclovirus (CyCV), chCyCV-SDAU-1, was identified in broilers with TVP. The complete chCyCV-SDAU-1 genome was verified via inverse PCR, was 1936 bp long, and consisted of Rep, Cp, and two intergenic regions. Phylogenetic tree analysis showed that chCyCV-SDAU-1 formed an independent branch with other cycloviruses. The homology of chCyCV-SDAU-1 with 20 others known cycloviruses was < 40%. Retrospective investigation showed that the CyCV infection rate in the broilers with TVP was 80% (16/20), while no CyCV was found in healthy chickens. In conclusion, a novel CyCV was identified in chickens with TVP, though its role in this disease is unclear.

## Introduction

The viral family *Circoviridae* are non-enveloped, spherical viruses with covalently closed, circular, single-stranded DNA (ssDNA) genomes. *Circoviridae* comprises two genera of circoviruses and cycloviruses (CyCVs), which have an ambisense genome organization containing two major inversely arranged open reading frames (ORFs) encoding replication-associated protein (Rep) and capsid protein (Cp) (1, 2). CyCV ORFs appear to be mirror images of those of the circovirus (1). Rep is the most conserved protein, which has motifs characteristic of proteins involved in rolling circle replication (RCR). Therefore, Cp is significantly divergent and is characterized by an N-terminal region that may provide DNA binding activity (3). A stem-loop structure containing a canonical non-anucleotide motif (TAGTATTAC) is located between the 5′ regions of Rep and Cp, which is the origin of DNA replication (1).

CyCV genomes were initially found in the feces of Pakistani children with and without acute flaccid paralysis (4) and were later discovered in stool samples from primates (humans and chimpanzees) and muscle tissue samples from other animals (camels, cows, goats, sheep, and chickens) (5, 6). These genomes have also been reported in other mammals (cats, bats, and squirrels), birds (ducks and wild birds), insects (dragonflies and cockroaches) and the environment (sewage) (7–14). Although the CyCV pathogenicity is unclear, CyCV genes have been found in serum, cerebrospinal fluid, and respiratory specimens from patients with paraplegia, acute central nervous system infections, and respiratory tract infections, respectively (15–18). To date, no cases of transmissible viral proventriculitis (TVP)-associated CyCV have been reported.

TVP, also known as malabsorption syndrome or runting-stunting syndrome, is characterized by poor growth, retarded feathering, diarrhea with undigested food, and increased mortality in broilers, broiler breeders and layer hens and has a negative economic impact on the poultry industry (19, 20). For decades, researchers have attempted to identify the TVP etiology. Many viral families have been implicated with TVP either alone or in combination, including *Adenoviridae, Reoviridae, Coronaviridae, Circoviridae, Anelloviridae, Astroviridae, and Picobirnaviridae* (21–28).

Chicken proventricular necrosis virus (CPNV) is a recently described birnavirus, which has been proposed to be the cause of transmissible viral proventriculitis (TVP). In the recent research, intranuclear inclusion bodies were observed in case of the submissions with TVP. The vast majority of these cases gave negative CPNV RT-PCR results, raising the question of whether another virus different from CPNV is responsible for some of these TVP-affected cases (29).

Here, we report the detection of a novel CyCV in broiler chickens with TVP from Weifang, China.

## Materials and Methods

In October 2018, a TVP outbreak occurred in 30-day-old commercial broilers on a farm in Weifang, China. All the same strains of commercial broilers were housed in six separate houses, and ~5,000 chickens were bred in each house. TVP broke out in forty-two chickens (0.94%) in one of the houses. Four days after the onset of sickness, twenty of forty-two (47%) chickens died, the remaining twenty-two chickens showed typical TVP symptoms, such as poor growth, retarded feathering and diarrhea with undigested food. Proventricular samples were collected from 20 of the dead broiler chickens and 10 of the healthy chickens in same flock, fixed in 10% neutral buffered formalin. TVP was diagnosed according to clinical symptoms and gross and histological lesions from the proventricular samples. These samples had pretested negative for pathogenic bacteria, avian leukosis virus subgroup J (ALV-J), Marek's disease virus (MDV), reticuloendotheliosis virus (REV), infectious bursa disease virus (IBDV), avian reovirus (ARV), chicken anemia virus (CAV), infectious bronchitis virus (IBV), chicken proventricular necrosis virus (CPNV), gyrovirus 3 (GyV3), and chicken circovirus (CCV). The primers for these pathogens were previously described (26). The PacBio-RSII sequencing platform (Novogene) was used to search proventricular viruses of TVP in chickens. Proventricular samples from diseased chickens were treated as previously described (27). BLASTx was used to translate sequence subreads and search for similar viral sequences. The complete CyCV genome was verified via inverse PCR. Two primer pairs for inverse PCR, CyCV-F1 (GTCTTTAGAGCGAACGG) and CyCV-R1 (GCTGTGGACGAAGGTAA) and CyCV-F2 (TACCGACTACAGGAGGAT) and CyCV-R2 (GTAATGTGGCTTCAAGAG), were used for the first and second PCR rounds, respectively. Samples were treated and amplified as previously described (26). Three repeat samples were tested. The PCR amplification products of the first and second rounds were connected to the T vector and spliced using SeqMan software after Sanger sequencing. CyCV ORFs were predicted using SnapGene software, and the secondary structure of the intergenic regions (IRs) was predicted using the Mfold program (30). Phylogenetic analysis was performed using MegAlign.

## Results and Discussion

**Figure 1:**
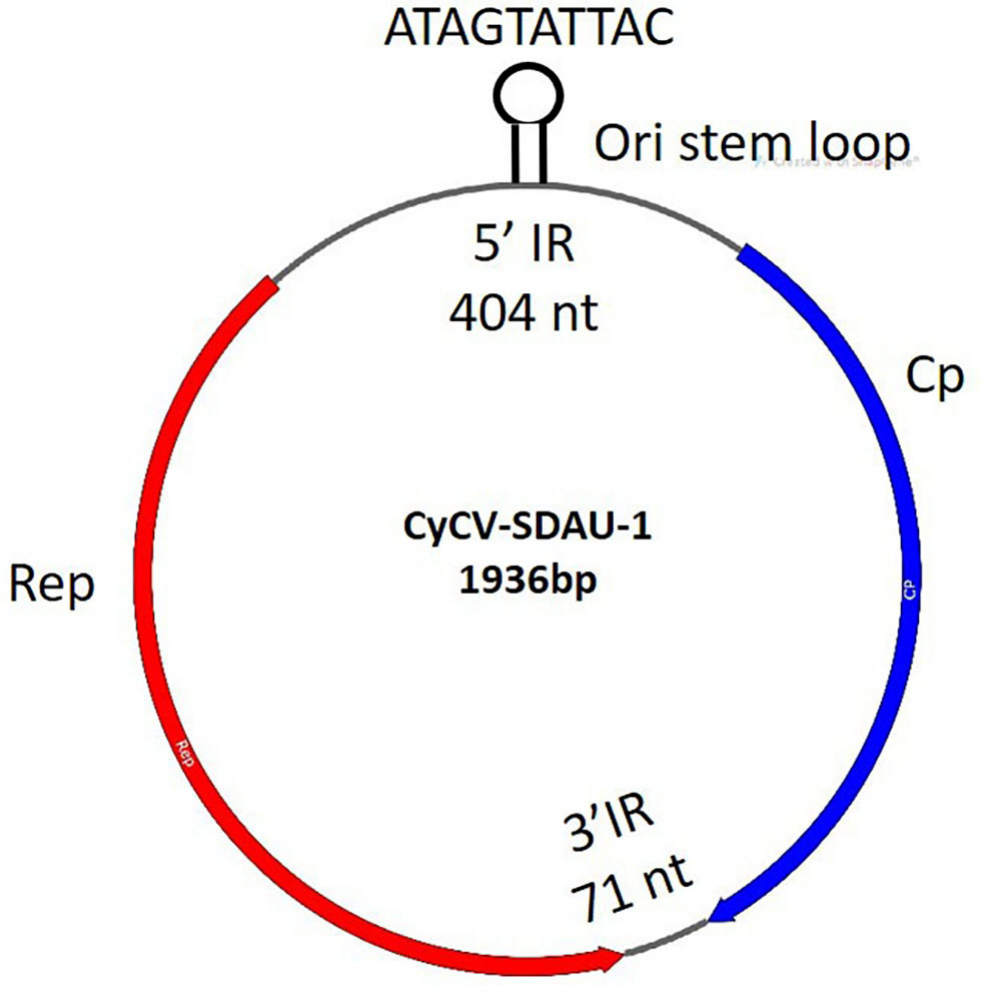
Gene structure comparison between CyCV-SDAU-1 and three other chicken cycloviruses. Compared with CyCV-SDAU-1, the other three cycloviruses showed circovirus characteristics.

TVP, characterized by proventricular enlargement, ingesta retention in the lumen, and fragility of the gastric isthmus, has become a major issue worldwide in avian flocks since the 1970s (31). TVP was diagnosed according to the presence of clinical runting-stunting symptoms, gross lesions in the swollen proventriculi, histological lesions in the proventriculi, including necrotic oxynticopeptic cells, lymphocytic infiltration, glandular hyperplasia, and metaplasia (Supplementary Figure 1). Because TVP mainly affected the proventriculus, proventricular samples were collected from TVP-affected chickens for viral detection. More than 40% of the cyclovirus (chCyCV-SDAU-1 [GenBank MN428468]) genome subreads were identified from 77,059 subreads using BLASTx. The identified chicken chCyCV-SDAU-1 was 1936 nt long, with 43.44% GC content, including the major ORFs for Rep (274aa) and Cp (211aa) and two non-encoded IRs (Figure 1). The chCyCV-SDAU-1 Rep contained motifs that differed from other CyCV RCRs and superfamily 3 (SF3) helicase motifs. The chCyCV-SDAU-1 Rep included only RCR motif I (SFTLLN) and SF3 helicase motif C (ITST), while Rep from other CyCVs includes RCR motifs I (FT[L/W]NN), II ([P/x]HLQG) and III (Y[C/l][S/x]K) and SF3 helicase motifs Walker-A (G[P/x][P/t][G/x]xGKS), Walker-B (uuDDF), and motif C (uTS[N/e]) (1). The chCyCV-SDAU-1 Cp is characterized by an N-terminal region rich in basic amino acids. The IRs were 404 nt long at the 5′ ends and 71 nt long at the 3′ ends of the major ORFs. The IR at the 5′ ends contained eight typical stem loops and a highly conserved ori stem loop (32 bp) marked by “ATAGTATTAC,” which slightly differed from those of other CyCVs (TAGTATTAC), at the apex of the stem loop structure (1). The IR at the 3′ ends contained a tandem combination of two stem loops.

**Figure 2:**
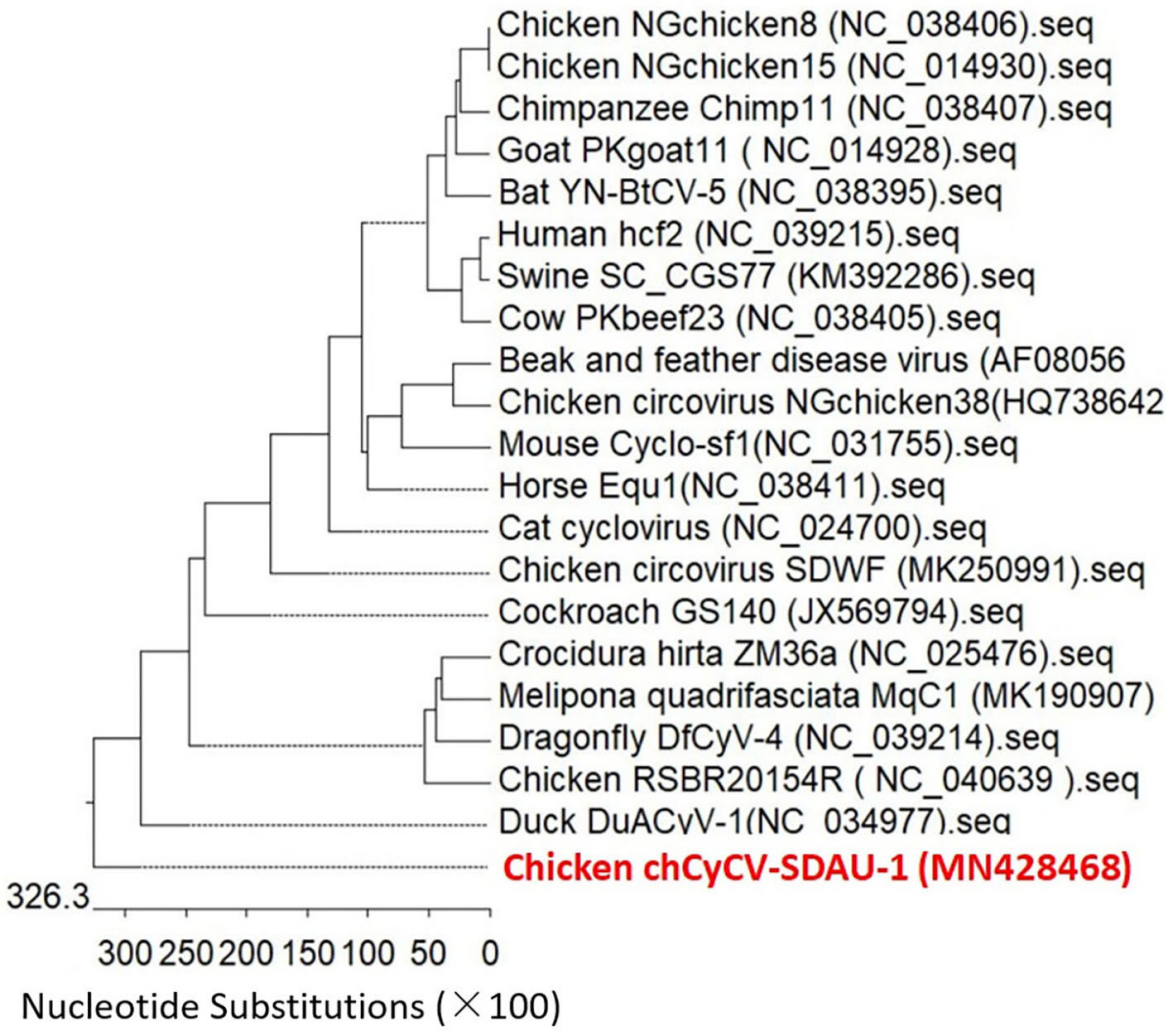
chCyCV-SDAU-1 gene structure. CyCV-SDAU-1 containing two major ORFs of the Rep and Cp proteins and two IRs.

**Figure 3:**
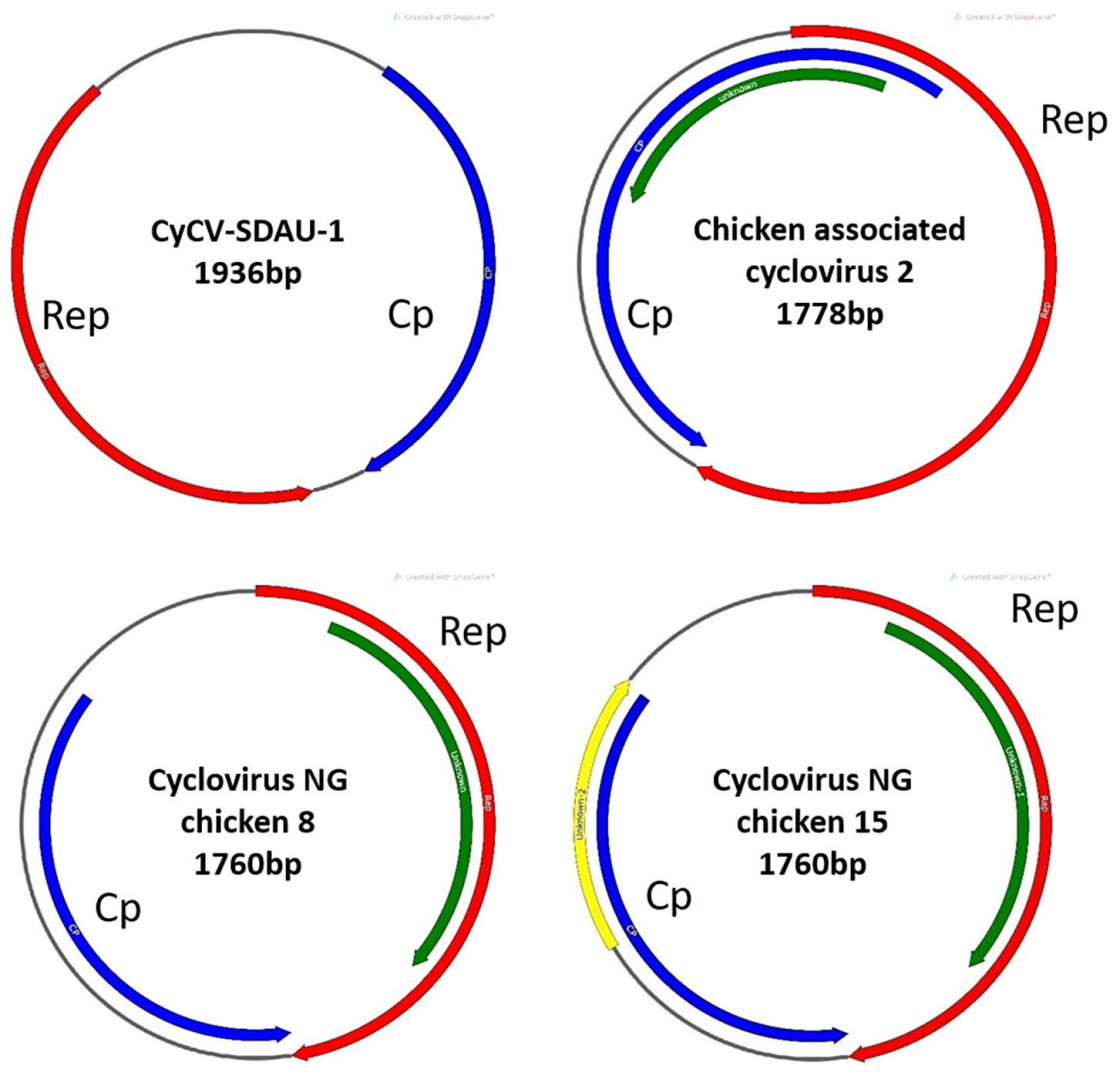
Phylogenetic analysis of whole-genome sequences of the CyCV-SDAU-1 based on nucleotide sequences. The phylogenetic tree showed that CyCV-SDAU-1 may be the origin of other cycloviruses or some circoviruses. The phylogenetic analysis was performed using MEGA 6.0 using the neighbor-joining method with 1,000 bootstrap replicates. Scale bars are proportional to the genetic distance. Bootstrap values > 70% are shown at each node. Red and bold indicate the CyCV-SDAU-1 strains.

Phylogenetic analysis confirmed that chCyCV-SDAU-1 differed from other known members of the CyCV genus (Figure 2). Our pairwise genetic analysis revealed that the entire chCyCV-SDAU-1 genome shared <40% nucleotide identity with the closest CyCV genome (Supplementary Figure 2). Several cycloviruses had been identified in chickens (6). However, further gene structure analysis revealed that these viruses were not CyCVs but circoviruses owing to the opposite Rep and Cp positioning (Figure 3). Thus, chCyCV-SDAU-1 is proposed as a prototype strain of CyCV in broilers. Interestingly, phylogenetic analysis of the CyCV genomes showed that all CyCVs except chCyCV-SDAU-1 and DuACyV-1 contained no clusters by the organism type from which they were identified. Furthermore, close genetic similarity of a subset of CyCVs replicating in distinct animal species indicated that chCyCV-SDAU-1 may be a prototype of other cycloviruses (Figure 2).

To determine the chCyCV-SDAU-1 prevalence in the TVP samples, nested and inverse PCR assays revealed that the chCyCV-SDAU-1 infection rate in the broilers with TVP was 80% (16/20). No chCyCV-SDAU-1 was found in healthy chickens, indicating that chCyCV-SDAU-1 might be associated with TVP. Although the novel CyCV was identified in TVP affected chickens, its pathogenicity, epidemiology, and public health effects remain unclear.

Applying viral metagenomics to characterize viromes has enabled identifying large-scale and highly diverse novel circular ssDNA viruses from different hosts and environments. To date, many ssDNA viruses have been identified in healthy hosts. Because of their ubiquitous distributions (32), ssDNA viruses have been identified in diseased hosts, but determining their pathogenicity is difficult. CyCV, a novel genus identified 10 years ago from the *Circoviridae* family, is associated with human acute flaccid paralysis (4), encephalitis, diarrhea (14), respiratory tract infections (15), paraplegia (17), acute central nervous system infections (18), and feline diarrhea (33). Here, a novel CyCV that might be associated with TVP in chickens was identified in broilers.

Despite efforts to understand the TVP pathogenesis, the causative agent remains unknown in some cases. In this study, we diagnosed TVP by its clinical symptoms, gross and histopathological lesions, and negative PCR results for known TVP-associated pathogens, including pathogenic bacteria, ALV-J, MDV, REV, IBDV, ARV, CAV, IBV, CPNV, GyV3, and CCV, we used the PacBio-RSII sequencing platform and inverse PCR to identify the possible pathogen. After subread splicing, structural prediction and phylogenetic analysis, we identified a dominant abundance of a novel cyclovirus.

Compared with other cycloviruses, the chCyCV-SDAU-1 gene structure had distinct features, including a lack of motifs II and III in Rep, a longer IR in the 5′ region of two ORFs, and a decamer (ATAGTATTAC) rather than a nonamer (TAGTATTAC) in the ori stem loop. In addition, the chCyCV-SDAU-1 sequence homology shared <40% identity with other CyCVs. However, although chCyCV-SDAU-1 might be associated with chicken TVP, assigning the isolate to a chicken origin is challenging. Thus, several properties of chCyCV-SDAU-1, including its pathogenicity, epidemiology and public health effects, require further investigation.

Given that there are 11 identified TVP-associated pathogens, the finding of yet another possibly associated with this syndrome is not compelling evidence for its role in TVP. In addition, this TVP outbreak might have another, unidentified cause that promotes shedding of this novel, perhaps asymptomatic CyCV in the affected chickens. We will try to prove the association between this novel CyCV and TVP in future studies by using a technique of *in situ* detection of the virus within the lesions.

In conclusion, a novel CyCV was identified in chickens with TVP, though its role in this disease is unclear.

## Data Availability Statement

The viral sequences are deposited in GenBank (MN428468). Other datasets used in the current study are available from the corresponding author on reasonable request.

## Ethics Statement

All the animal experiments in this study were carried out in accordance with the recommendations of the Shandong Agricultural University Animal Care and Use Committee. The protocol was approved by the Ethics Committee for Animal Experiments of the Shandong Agricultural University, and the approval number is SDAU-2019-21. All diseased chickens were sent to our lab by the owner and permitted to diagnose the disease.

## Author Contributions

ZC designed the experiment and wrote the manuscript. TY and GL did all experiments. DZ analyzed the clinical cases and performed the necropsies and the pathological examinations. All authors contributed to the article and approved the submitted version.

## Conflict of Interest

The authors declare that the research was conducted in the absence of any commercial or financial relationships that could be construed as a potential conflict of interest.

## Abbreviations

TVP: transmissible viral proventriculitis
MAS: malabsorption syndrome
RSS: runting-stunting syndrome
ssDNA: single-stranded DNA
ORF: open reading frame
Rep: replication-associated protein
Cp: capsid protein
RCR: rolling circle replication
SF3: superfamily 3
ALV-J: avian leukosis virus subgroup
MDV: Marek's disease virus
REV: reticuloendotheliosis virus
IBDV: infectious bursal disease virus
ARV: avian reovirus
CAV: chicken anemia virus
IBV: infectious bronchitis virus
CPNV: chicken proventricular necrosis virus
GyV3: Gyrovirus 3
CCV: chicken circovirus
chCyCV-SDAU-1: chicken cyclovirus SDAU-1 strain.
