# Supplementary figures and images for: Novel Cyclovirus Identified in Broiler Chickens With Transmissible Viral Proventriculitis in China
Source: Front Vet Sci. 2020 Sep 29;7:569098. doi: 10.3389/fvets.2020.569098 (PMC7550471; doi:10.3389/fvets.2020.569098)

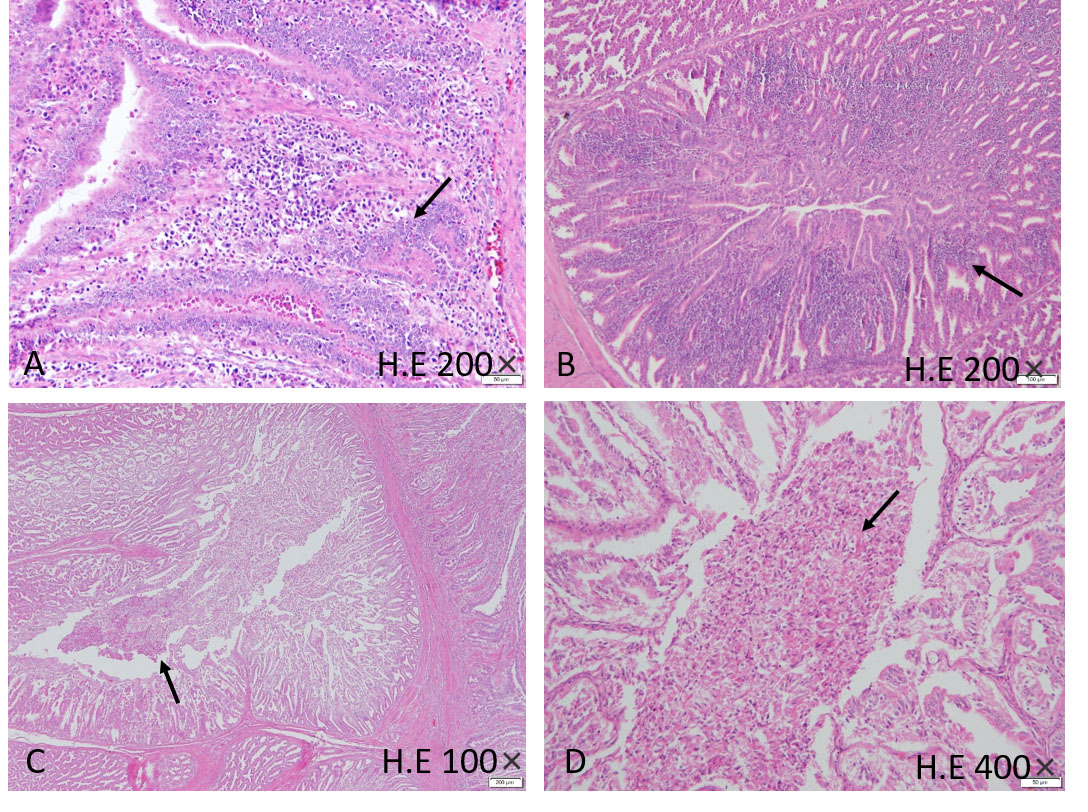

Supplement: Supplementary file 1 [file Image_1.JPEG]

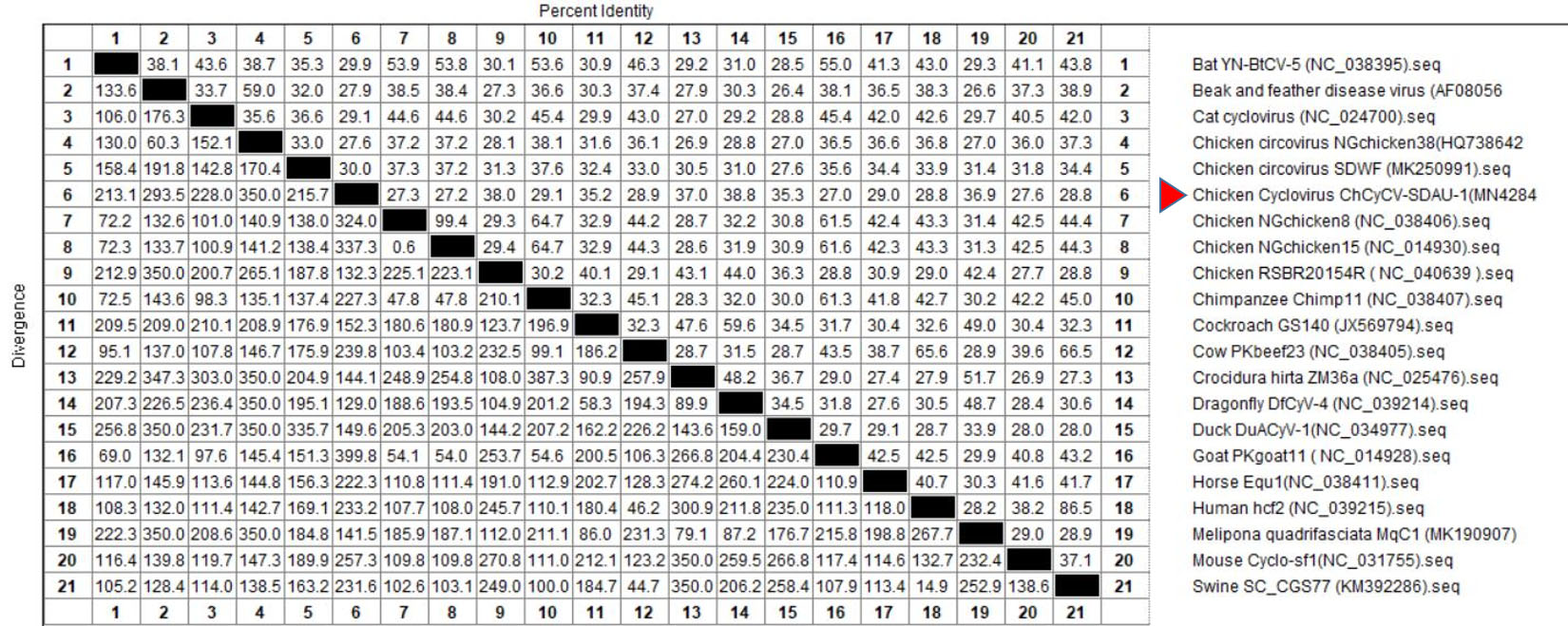

Supplement: Supplementary file 2 [file Image_2.JPEG]
