# Supplementary material for: Novel Cyclovirus Identified in Broiler Chickens With Transmissible Viral Proventriculitis in China
Source: Front Vet Sci. 2020 Sep 29;7:569098. doi: 10.3389/fvets.2020.569098 (PMC7550471; doi:10.3389/fvets.2020.569098)
Supplement: Supplementary file 3 [file Data_Sheet_1.docx]

**Supplementary Figure 1**

Histological lesions of chicken cases infected by TVP. (A) Glandular hyperplasia and metaplasia. (B) Proventricular interstitial lymphocytic infiltration. (C) At low magnification, necrotic oxynticopeptic cells. (D) At high magnification, necrotic oxynticopeptic cells.

**Supplementary Figure 2**

Pair distance of CyCV-SDAU-1. pair-wise genetic analysis revealed that CyCV shared less 40% nucleotide identity over the entire genome to the closest cyclovirus genome.
